# Crucial Role of Silica-Alumina Binder Mixtures for Hydrocarbon Cracking with ZSM-5 Additives

**DOI:** 10.1021/acsomega.2c05003

**Published:** 2022-11-29

**Authors:** Liane A. Haufe, Vladislav Timoshev, Markus Seifert, Oliver Busse, Jan J. Weigand

**Affiliations:** Faculty of Chemistry and Food Chemistry, Chair of Inorganic Molecular Chemistry, Technische Universität Dresden, Mommsenstraße 4, Dresden 01069, Germany

## Abstract

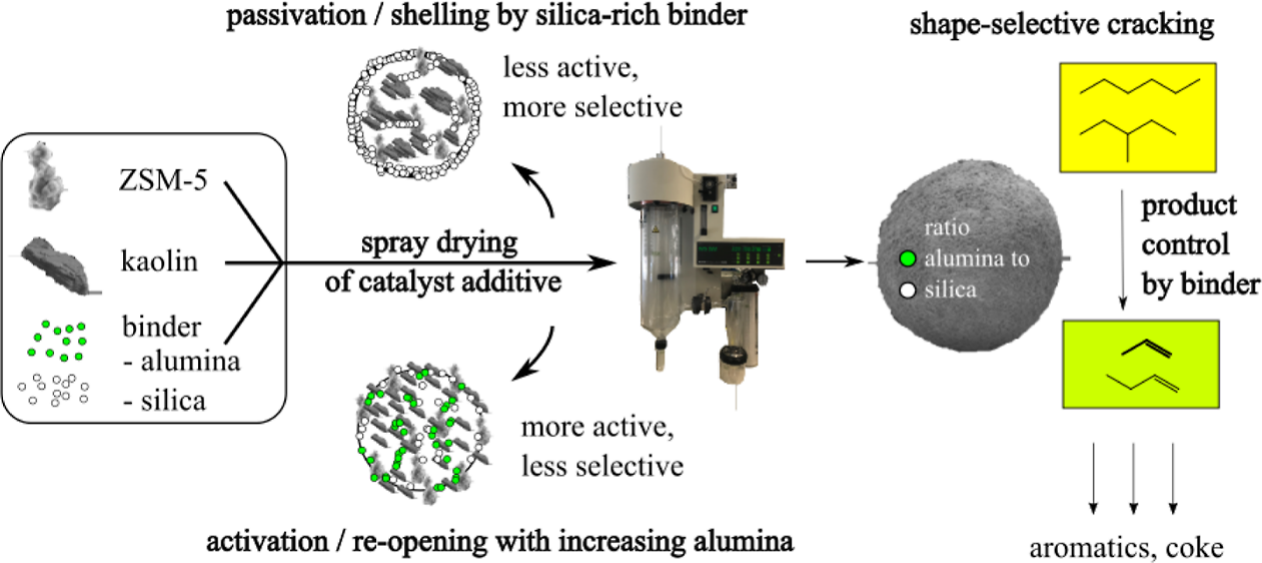

Alumina-containing binders are widely used for the binding of catalyst particles by spray drying and calcination. As a part of the active matrix, they contribute to the catalytic performance of the resulting catalyst grain during hydrocarbon cracking. In this study, correlations are investigated using different compositions of Al- and Si-based binders (AlCl_3_ and colloidal silica) together with kaolin as a filler and ZSM-5 zeolite as an active compound. It was demonstrated that the conversion of a 50:50 hexane mixture, the selectivity toward unsaturated hydrocarbons, and the shape-selective conversion of the hexane feed are highly dependent on the amount and distribution of alumina in binder formulations. While silica species are distributed near the outer shell of catalyst grains, the alumina species are distributed evenly as an adhesive between the catalyst compounds ZSM-5 and kaolin. An optimum amount of alumina in binder formulations results in an increasing conversion of hydrocarbon feedstock due to optimum in active-site accessibility but only a slight decrease in shape-selective properties compared to pure ZSM-5, resulting in an optimum yield of light olefins, especially propylene.

## 1 Introduction

**Figure 1:**
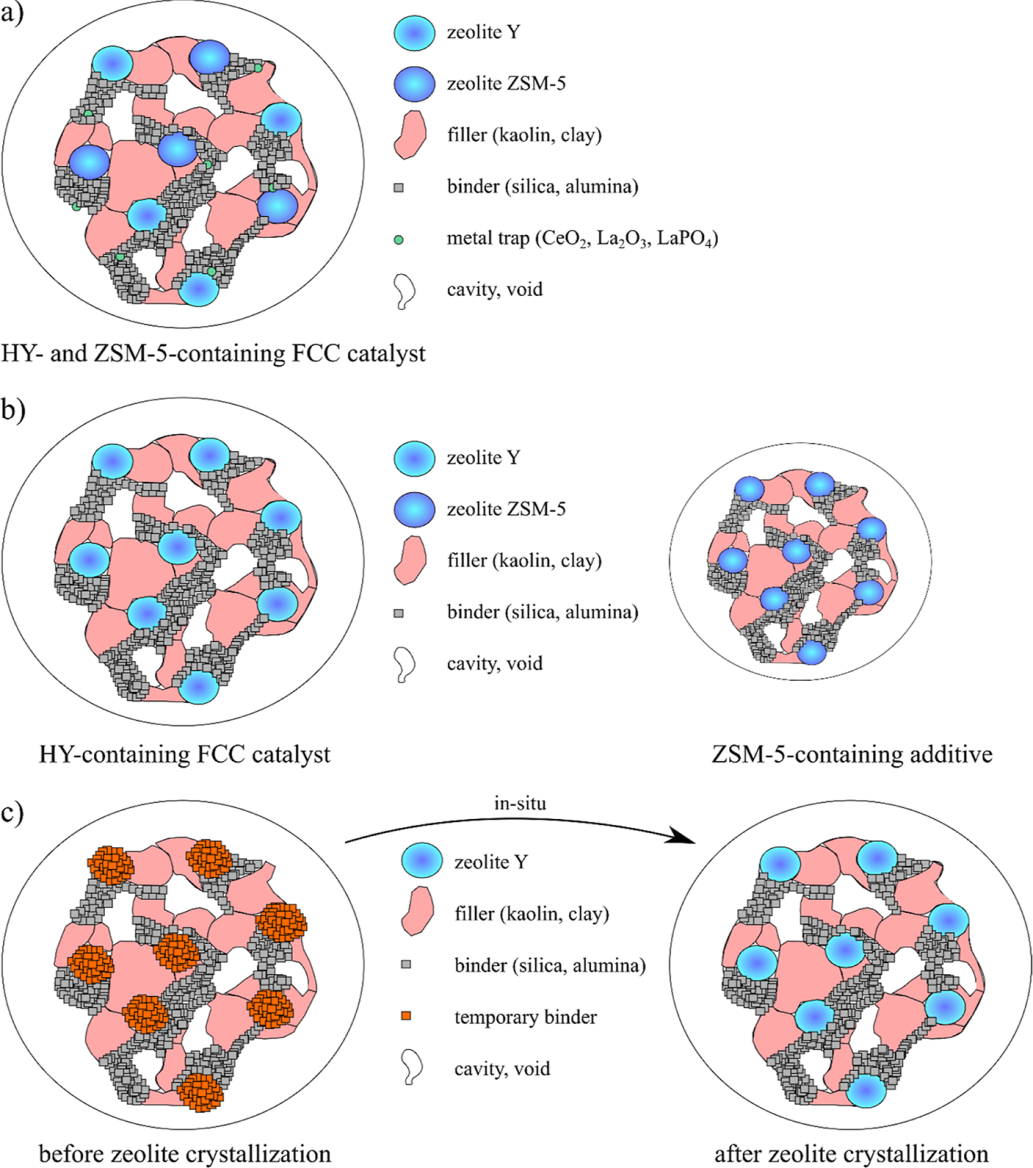
Pictograms of catalyst grains for catalytic cracking produced from different synthesis strategies: (a) synthesis of catalyst grains from spray drying of individual components in complete grains; (b) synthesis of different catalyst and additive grains from individual components by spray drying; and (c) in-situ production of different compounds after spray drying of the catalyst body.

Catalysts used for catalytic cracking are usually composites made of different components. These are often an active component, for example, zeolites, a filler such as kaolin, different porogens, a binder, and few different promotors and additives. In the case of cracking catalysts, there are three general options for production:^1^ The first is the combination of all separate components in one particle via spray drying, which provides a straight-forward synthesis route after optimization but lacks flexible catalyst adjustment to varying feed qualities^1−4^ (Figure 1a). The second is the use of different individual grains for the fine-tuning of additive addition, depending on a flexible change in the hydrocarbon feed composition, which hides the challenge of adjusting the thermal and rheological properties of all particles individually and in combination^1,2,5^ (Figure 1b). The third is the in-situ production of different components during or after catalyst shaping for enhanced mechanical and transport properties, which again can be used as a single grain or with different additive particles^1^ (Figure 1c).

In the case of hydrocarbon cracking with ZSM-5 for enhanced olefins (at higher temperatures) or gasoline production, the use of separate additive grains is more common, whose compounds are summarized in the easiest way with active zeolite (ZSM-5), a filler such as kaolin, and a binder. Since the binder can be defined as part of the active matrix, it has a major impact on the catalytic properties as well as the hydrothermal and mechanical stabilities of the formulated grains and their coking behavior.^2,6^ The main activity of the catalyst is therefore determined by the zeolite and the binder formulation used, although acidic alumina binders, in particular, can lead to increased cracking activity of the formulated catalyst.^6,7^ The filler (e.g., clay or, in particular, kaolin) has different functions: it acts as a (catalytically) inert filler material to enhance the size of the grains, has suitable rheologic properties, and supports mechanical stability. Sometimes, clay is used as a basis in-situ to grow active zeolites after spray drying (see Figure 1c). Moreover, the reduction in density of active material (e.g., zeolite) and the formation of additional slit pores reduce the risk of hotspots and, thus, cause overabundant coking or structural break of the grains.^5^

The shape selectivity of the formulated catalysts can be tested via constraint index (CI) method. The simultaneous feeding of linear alkane *n*-hexane and bulkier isomer 3-methylpentane shows the favored starting material for cracking over the catalyst. The ratio of the conversion rate of both molecules is defined as the CI.^8,9^ Maximizing the yield of desired products and the lifetime of a catalyst is an important aspect for the development of more sustainable catalytic cracking of hydrocarbons.^10^ As olefins have become important building blocks,^2,11^ the demand for these important hydrocarbons is continuously rising.^12^

The focus of this work is on spray drying and the formulation of additives containing ZSM-5 for the catalytic cracking of low-boiling hydrocarbons. In order to compare the dependence of conversion and selectivity toward olefins, two different binder types, AlCl_3_ and silica (in the form of LUDOX), were elected for investigation via CI since the type of binder has a major influence on the catalytic properties.

## 2 Materials and Methods

### 2.1 Utilized Raw Materials

All the chemicals were used as supplied without further purification. The chemicals used for the preparation of catalyst additives were NH_4_-ZSM-5 (CBV 5524G, Zeolyst, SiO_2_/Al_2_O_3_ = 50, Si/Al = 25), kaolin (Sigma-Aldrich), aluminum chloride hexahydrate (AlCl_3_·6H_2_O, Alfa Aesar, 99%) and LUDOX HS-40 (SiO_2_·*x*H_2_O, 40% SiO_2_, Sigma-Aldrich). OlefinsUltra (Grace) was used as a reference material for catalytic tests. The chemicals used for catalytic testing were 3-methylpentane (C_6_H_14_, Acros Organics, 99%) and *n*-hexane (C_6_H_14_, VWR, ≥97%). Sample digestion for subsequent elementary analysis by ICP-OES required hydrochloric acid (HCl, VWR, 37%), nitric acid (HNO_3_, Carl Roth, 69%, supra-quality), hydrofluoric acid (HF, Merck Millipore, 40%), and boric acid (B(OH)_3_, Alfa Aesar, 99.99%).

### 2.2 Sample Preparation

Zeolite (CBV 5524G) was calcined at 540 °C for 6 h under air flow to obtain the catalytically active form H-ZSM-5. The zeolite and filler (kaolin) of the additives were sonicated in an aqueous suspension for 8 min (400 W max. power, output control 70%, duty cycle 35%) to break up the agglomerates of ZSM-5 and also to reduce the size of the kaolin particles. The binder was then added, and the mixture was stirred at 500 rpm for 15 min before being spray dried using a B290 Advanced from Büchi (ϑ_inlet_ = 210 °C, *V̇*_drygas_ = 35,000 L·h^–1^, *V̇*_feed_ = 1.38 L·h^–1^, *V̇*_spraygas_ = 538 L·h^–1^). The spray drying process typically took 10 min for each sample, except one upscaling (“_S”), which took 30 min under similar conditions to reach a three times higher amount of the final product. After this procedure, the spray-dried product was calcined at 650 °C for 8 h under air flow (30 L·h^–1^).

For catalytic testing in a laboratory-scale test reactor (fixed bed), the samples were pressed to pellets, crushed, and sieved to obtain a fraction of particles in the range of 315–400 μm to ensure reproducible flow conditions. Smaller fractions tend to leave the catalyst bed and block the reactor, while excessively bigger grains have shown low activity, which indicates additional transport restrictions and short flow.

All samples were prepared in a 30 wt % slurry. Since ZSM-5 and kaolin, as the solid components of the slurry, are mainly responsible for coagulation, their amount of substance was fixed at 1.32 and 9.30 mol, respectively. Only the soluble components of the formulation (namely AlCl_3_ and LUDOX) were varied. The nomenclature was chosen as: A_*n*, where *n* is the amount of substance of AlCl_3_ and the rest of the binder formulation is LUDOX HS-40. The index *S* means that a scale up by a factor of 3 was performed in the spray drying process by a three-times longer spray process under similar process conditions. As another reference for catalytic tests only, the sample kaolin_A_10 was prepared analogous to all other samples despite the fact that the amount of ZSM-5 was replaced with additional kaolin. The detailed compositions of the samples are summarized in Table 1.

**Table 1:** Composition of Additives for all Samples in this Study

sample name	A_10	A_7.5	A_5	A_5_S	A_2.5	A_0	kaolin_A_10
binder	AlCl_3_	AlCl_3_ and LUDOX	AlCl_3_ and LUDOX	AlCl_3_ and LUDOX	AlCl_3_ and LUDOX	LUDOX	AlCl_3_
n(ZSM-5) [mmol]	1.32	1.32	1.32	1.32	1.32	1.32	0.00
n(kaolin) [mmol]	9.30	9.30	9.30	9.30	9.30	9.30	24.80
n(AlCl_3_) [mmol]	10.06	7.55	5.03	5.03	2.51	0.00	10.06
n(LUDOX) [mmol]	0.00	13.31	26.63	26.63	39.94	53.26	0.00
Si/Al_binder_ [molar]	0	1.84	5.51	5.51	16.53	→∞	0

### 2.3 Characterization of Slurries

To ensure successful spray drying, the ζ-potential was investigated for all suspensions at different pH values from 3 to 6. A Zetasizer from Malvern Panalytical was used for this purpose. At a constant temperature of 25 °C, 10 to 100 replicate measurements were accumulated and pooled to eliminate noise.

### 2.4 Sample Characterization

The specific surface areas of the samples are quantified using a Surfer from Thermo Fisher Scientific. For this purpose, the samples are dried under vacuum at 250 °C for 8 h to remove all adsorbed gases and residual water. Samples are then cooled in liquid nitrogen to record physisorption isotherms. The specific surface area was calculated using the BET equation^13^ in a linear range of adsorption isotherms from *p*/*p*_0_ = 0.001 to *p*/*p*_0_ = 0.04 (<2 nm pore width).^14,15^ The mesoporous specific surface area was calculated using the Barrett–Joyner–Halenda (BJH) method^16^ with the standard adsorption isotherm of Harkins and Jura^17^ in the range of the adsorption isotherm from *p*/*p*_0_ = 0.39 to *p*/*p*_0_ = 0.96 (2–50 nm pore width).^14,15^

Temperature-programmed desorption of ammonia (TPD) is an important method for studying the acidic properties (amount and strength of acid sites) of samples. In this work, a TPDRO 1100 instrument from Thermo Fisher Scientific equipped with a thermal conductivity detector was used. For the measurements, 150 mg of the samples were dried under argon flow for 2 h at 250 °C. Adsorption of ammonia is performed at 120 °C for 10 min. For desorption, the sample chamber is purged with helium for 3 h, followed by a heating step to 550 °C (10 K·min^–1^) with a dwell time of 1 h. The time-dependent temperature profile is shown in the Supporting Information (Figure S1 and Table S1). The acidity of strong acid sites was determined in the temperature range of 315–515 °C.

SEM images were recorded using a SU8020 scanning electron microscope (Hitachi) equipped with a triple detector system for secondary and backscattered electrons (*U*_a_ = 2 kV). The dried samples (200 °C) were fixed with carbon adhesives on an aluminum stamp. To avoid any charge-up and chemical alteration during the measurements, the surfaces of the samples were coated with gold in an automatic rotary-pump coating system (Quorum Q150R ES).

For EDX line mapping experiments, samples were dried at 200 °C overnight in an oven. An epoxy resin, PUK, was prepared to ensure a plain surface after sawing through the particles and polishing. The PUK was fixed with carbon adhesives on an aluminum stamp, and the charge-up was minimized using adhesive carbon tape and sample coating with gold (Quorum Q150R ES). The samples were dried in vacuum for 24 h. For the measurement, a voltage of 20 kV was used at a magnification of 3,500. Only the elements Si, Al, and Au were analyzed by their respective scattering; other elements such as Na, K, or C from resin and the underlying carbon pad were excluded to enhance visibility by a lower number of graphs in each diagram.

Static laser scattering on a Bettersizer S3 Plus from 3P Instruments was used to study particle size distribution. To disperse the samples in water, they were stirred at 2,000 rpm for 3 min and agitated using ultrasonic power of 50 W (38 kHz). The particle size distribution was calculated using Mie theory.^18^

The elemental composition of the samples was verified by ICP-OES. For this purpose, about 50 mg of each sample was dissolved in a mixture of 1 mL of hydrofluoric acid (40%), 2 mL of hydrochloric acid (37%), and 3 mL of nitric acid (69%). After a waiting period of 1 h, the remaining fluorides were quenched with 10 mL of saturated boric acid (170 °C) in a MARS 6 CEM microwave. The quantitative determination of elements was carried out with an Optima 2000 DV by PerkinElmer. Calibration was performed with solutions of the respective diluted ion standards from PerkinElmer. The wavelengths used for this analysis were 237.313, 394.401, and 396.153 nm for Al, 212.412 and 251.611 nm for Si, 334.940, 238.204, and 259.939 nm for Fe, and 334.940 and 337.279 nm for Ti.

Catalytic testing was performed in a tubular fixed-bed reactor made from stainless steel. Due to the need to reach a sufficient level of conversion for all active samples (40–70%), only 2 g of the sieved catalyst sample were installed between quartz wool supports. The reactor was heated up to 350 °C under nitrogen flow. The samples were calcined for 4 h to remove residues of water and adsorbed components. A carrier gas flow of 3 L·h^–1^ (GHSV = 1185 h^–1^) for the reaction and a mixture of 50 wt % *n*-hexane and 50 wt % 3-methylpentane with a flow of 3.3 g·h^–1^ (WHSV = 1.65 h^–1^) at 500 °C reaction temperature were chosen. Based on these starting materials, the evaluation of the shape-selective cracking via the catalyst additives was possible using the CI test method:^9,19^
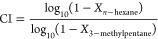
1

CI...constraint index
*X*_*n*-hexane_...conversion of *n*-hexane
*X*_3-methylpentane_...conversion of 3-methylpentane

These conversions were analyzed for 7 h time on stream (ToS) via a chromatographic analysis of the gaseous product phase at a Clarus 590 GC with a HP-1 100 m column, equipped with a flame ionization detector (FID) using a modified DHA method (detailed hydrocarbon analysis) shown in Table S2.^20^ A mass balance, including a gas phase and coke, was used for quantification. The amount of coke was determined by thermogravimetric analysis (TGA) using a Mettler Toledo TG50. About 20—30 mg of coked samples were heated with a linear temperature profile (10 K·min^–1^ from 35 to 850 °C, 20 mL·h^–1^ dried air). The amounts of coke on the catalysts after 7 h ToS including their fraction to the complete mass balance is listed in Supporting Information Table S3 for each test. However, the contribution to total hydrocarbon balance is always low (<0.1 wt %).

Diffractograms of the starting materials as well as the spray-dried products were recorded with a STADI P powder diffractometer (STOE & Cie.) using a DECTRIS MYTHEN 1K detector with Cu K_α,1_ radiation (λ = 0.154059 nm, curved Ge single-crystal monochromator). The step width was chosen to be 0.015° 2θ.

## 3 Results and Discussion

### 3.1 Properties of Slurries

**Figure 2:**
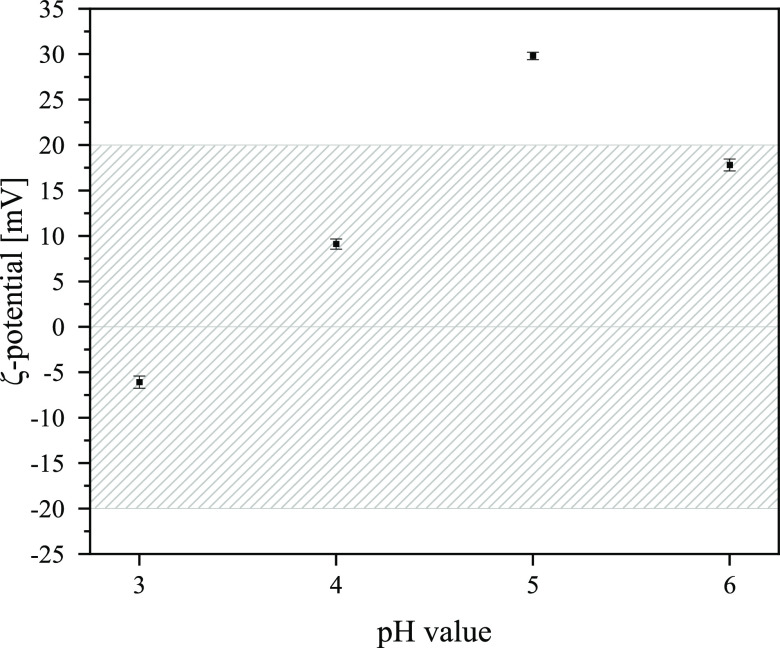
Dependence of the ζ-potential on the pH value of slurries represented by sample A_10 including error bars.

The ζ-potential of component mixtures for spray drying strongly depends on the pH value of the suspensions and is illustrated in Figure 2.

The profile in Figure 2 can be explained by different Al species that dynamically transform into each other by changing the pH value. At low pH values of 3 and below, aluminum(III) hexahydrate ions are the predominant species. The addition of the base and a consequent increase in pH results in an exchange of protons and water molecules between the inner coordinating aqua ligand sphere and the surrounding external water molecules.^21^

Consequently, the complex ion is hydrolyzed. The charge of the ion changes as a result, and so does the ζ-potential. With further increase in the pH, the complex mononuclear aluminum(III) ions agglomerate to form polynuclear species such as the Keggin-type cation [Al_13_O_4_(OH)_24_(H_2_O)_12_)]^7+^ and many oligomers in between.^22^

According to Vallar et al.,^23^ the ζ-potential indicates good stability or the possibility for suspensions to form agglomerates. Below a |ζ| of 20 mV (represented by the gray bar in Figure 2), suspensions are not stable and tend to form agglomerates. This phenomenon is important for successful spray drying because the components of the slurries must agglomerate during the spray drying process to produce uniformly sized, spherical catalyst grains.

All suspensions show low values for |ζ| at pH 3 and 4. Since AlCl_3_ and SiO_2_ in aqueous suspensions show pH buffering behavior at these pH values, no adjustment of the pH was necessary. All AlCl_3_-containing samples (A_10, A_7.5, A_5, A_5_S, and A_2.5) were spray dried at a pH of 3, and only the LUDOX-containing sample (A_0) was spray dried at pH 4. For further details, all ζ-potential values are summarized in Supporting Information Table S4.

### 3.2 Comparison of Grains after Spray Drying

**Figure 3:**
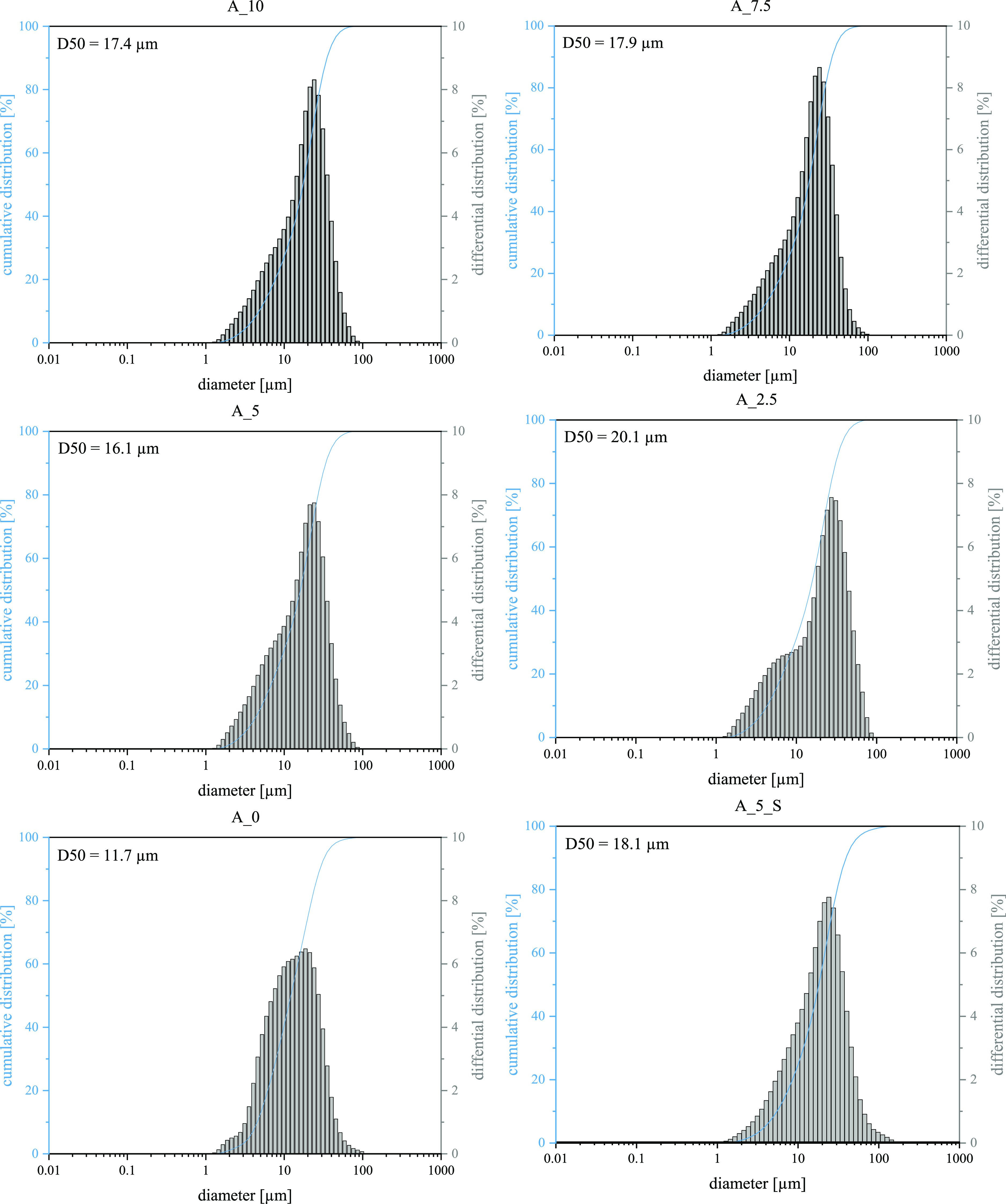
Particle size distribution of spray-dried grains after calcination.

Quantification of the particle size distribution of the spray-dried catalyst grains reveals that although the average size (value of D50) is almost the same for all AlCl_3_-containing samples, the distribution shows a different behavior. At high alumina binder content, the maximum particle size is smaller compared to a high silica binder content. As the proportion of LUDOX in the formulation increases, the particle size distribution becomes broader. A second fraction of particles of smaller size is represented by a forming shoulder, which is shown in Figure 3. This indicates a secondary aggregation of silica species to smaller particles.

**Figure 4:**
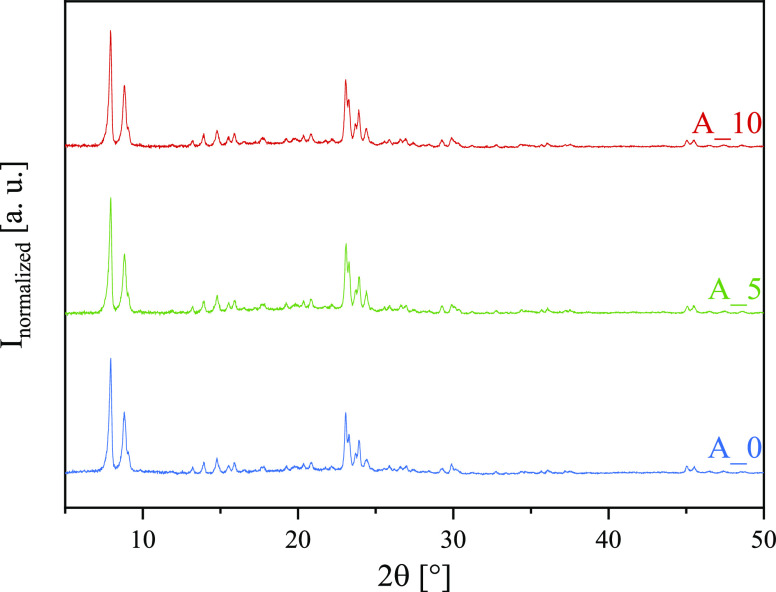
X-ray diffraction patterns of spray-dried additives A_0, A_5, and A_10.

The X-ray powder diffraction patterns show that only reflexes of the used ZSM-5 are present in all samples. No other dominant phases are formed, and the reflexes caused by the almost amorphous kaolin (metakaolin) are barely visible. The diffraction patterns for the representative samples A_0, A_5, and A_10 are displayed in Figure 4, and the diffraction patterns of the starting materials as well as the other samples can be found in Supporting Information Figure S2.

The compositions of the synthesized additives were analyzed by ICP-OES for the content of silicon, aluminum, iron, and titanium (see Table 2). As expected, the aluminum content depends on the amount of AlCl_3_ in the binder formulation, while the amounts of zeolite and filler remain constant. Similarly, the silicon content depends on the amount of LUDOX (colloidal silica) in the binder formulation. The impurities measured (iron and titanium) were caused by kaolin and are therefore in the same range for each sample.

**Table 2:** Elemental Composition of Al, Si, Fe, and Ti from ICP-OES Analysis

sample name	A_10	A_7.5	A_5	A_5_S	A_2.5	A_0	ZSM-5	kaolin
ω(Al) [wt %]	10.07 ± 0.03	9.01 ± 0.17	7.97 ± 0.37	8.23 ± 0.06	7.88 ± 0.04	6.69 ± 0.02	1.42 ± 0.01	20.70 ± 1.09
ω(Si) [wt %]	33.70 ± 0.70	34.15 ± 0.30	34.61 ± 0.50	35.20 ± 0.60	38.26 ± 0.20	36.26 ± 0.70	41.57 ± 0.90	22.27 ± 0.90
ω(Fe) [wt %]	0.20 ± 0.02	0.18 ± 0.01	0.17 ± 0.02	0.17 ± 0.00	0.18 ± 0.00	0.17 ± 0.01	0.02 ± 0.00	0.50 ± 0.02
ω(Ti) [wt %]	0.11 ± 0.01	0.10 ± 0.00	0.09 ± 0.01	0.11 ± 0.00	0.10 ± 0.01	0.10 ± 0.03	0.01 ± 0.00	0.33 ± 0.01

**Figure 5:**
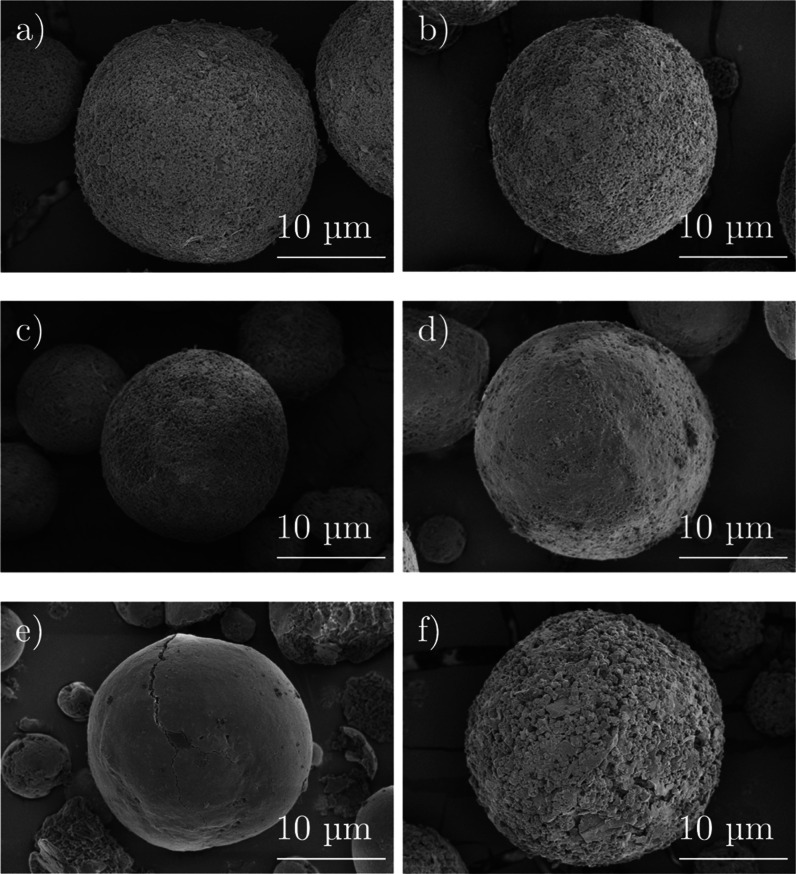
SEM pictures with a magnitude of 3500 for samples (a) A_10, (b) A_7.5, (c) A_5, (d) A_2.5, (e) A_0, and (f) A_5_S.

The following Figure 5 depicts the morphology of the spray-dried grains of ZSM-5 additives.

**Figure 6:**
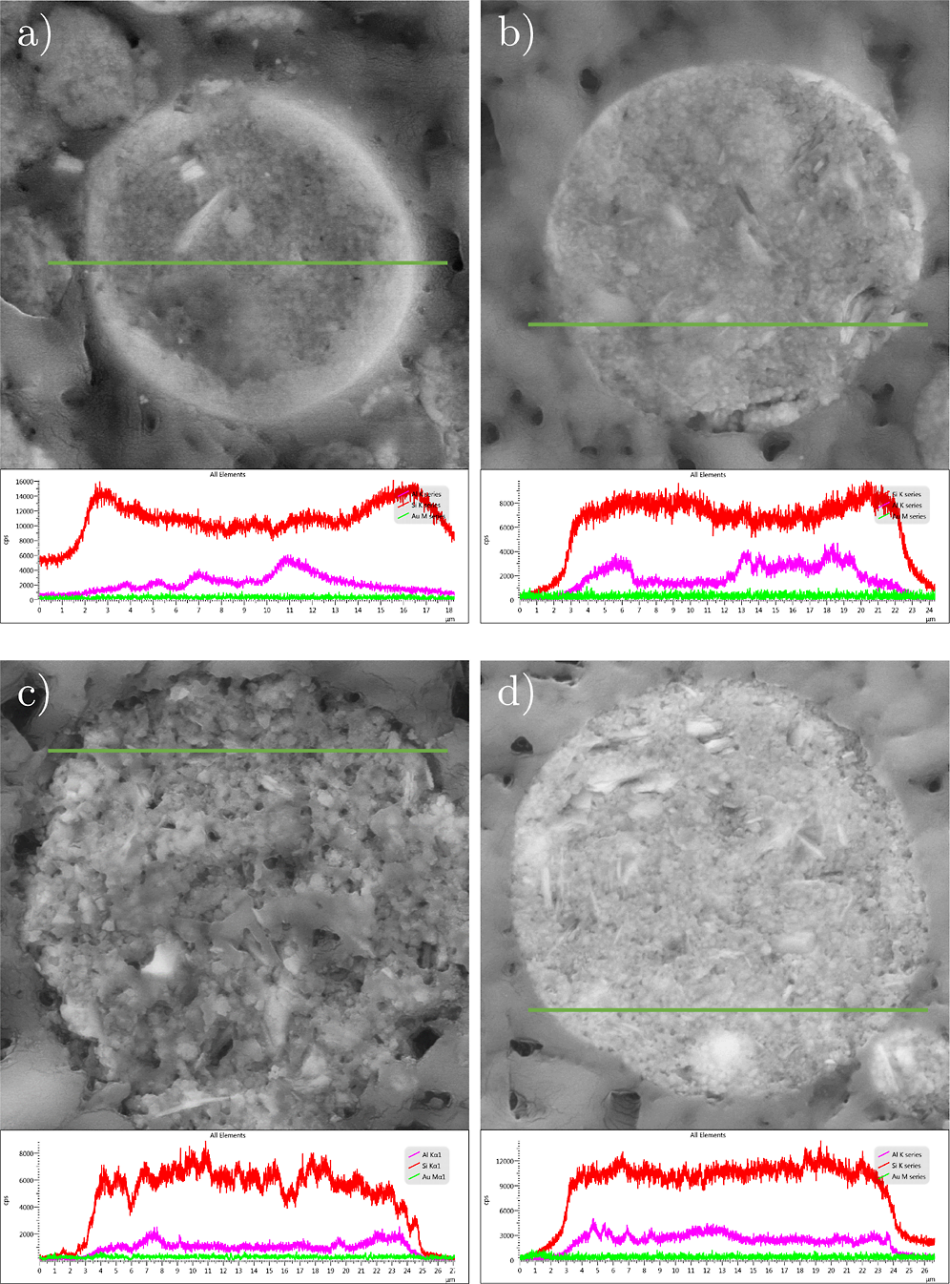
EDX line mapping for Si and Al of samples (a) A_0, (b) A_5, (c) A_5_S, and (d) A_10 (magnification 3,500, green line: measuring line, red/upper graph: Si K series, pink/middle graph: Al K series, and green/lower graph: Au M series).

The SEM images show spherical shapes for all samples. In addition, the surfaces of the particles appear rough with an increasing amount of alumina in the binder formulation. A deeper look into silicon and aluminum distribution (Figure 6) reveals that the silica binder generally forms a complete silicon-rich outer shell around the particles, pushing aluminum-rich species (e.g., zeolite) to the particle center. In contrast to this, an additional alumina binder provides more alumina and zeolite species distributed over the external grain surface. Consequently, alumina tends to be distributed more homogeneously over the other particle compounds. This suggests that there is a stronger interaction between the polar alumina binder and the other (polar) components than with the less polar silica binder. A comparison with particle size analysis (Figure 3) supports the idea that higher amounts of silica binder in the absence of alumina show less interaction with other catalyst components, which leads to an increasing agglomeration of smaller particles of silica with rising content of the LUDOX binder in the slurry for spray drying.

**Figure 7:**
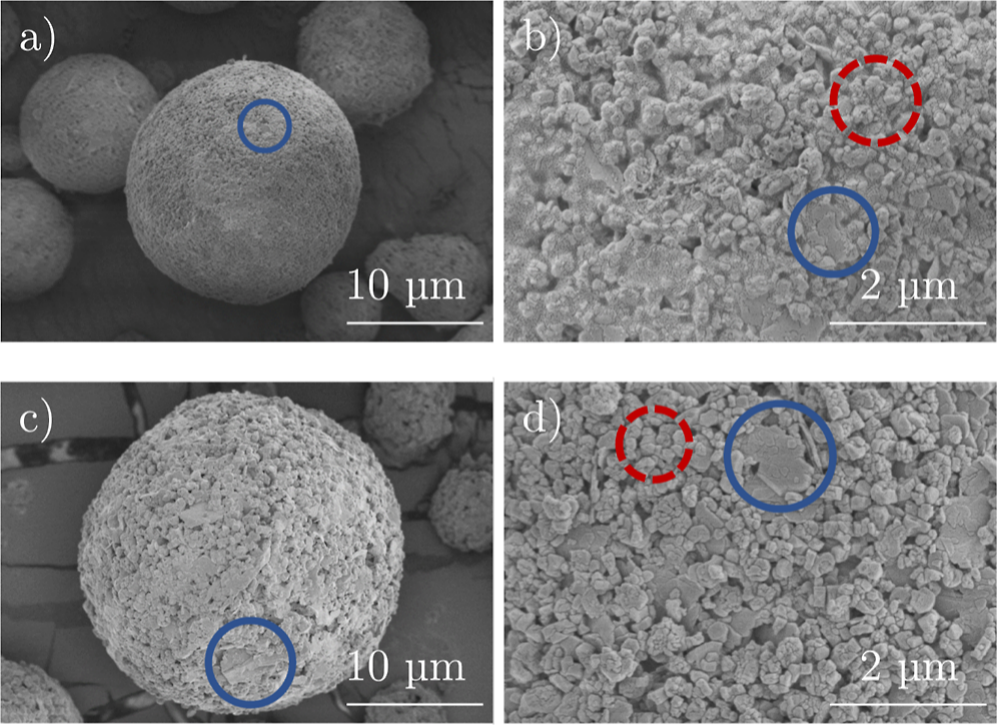
SEM picture of samples (a,b) A_5 and (c,d) A_5_S (magnifications 3,500 and 20,000): mark-up of differently sized (blue) kaolin platelets and (dotted red) ZSM-5 particles.

Although samples A_5 and A_5_S have almost the same composition, the SEM images show different morphology. In Figure 5c, kaolin platelets are smaller than the kaolin platelets of the scale-up sample shown in Figure 5f. This indicates that the ultrasonic treatment must be prolonged when larger amounts of slurry are used. A higher magnification of the spray-dried catalyst particles A_5 and A_5_S shows different sizes of kaolin platelets, which are depicted in Figure 7.

**Figure 8:**
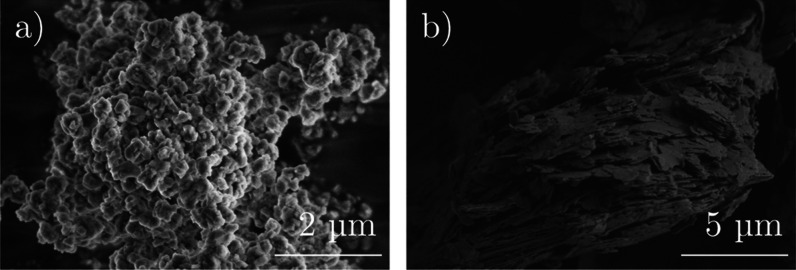
SEM pictures of commercial (a) H-ZSM-5 (magnification 20,000) and (b) kaolin (magnification 10,000).

A closer look at the morphology of the starting materials is shown in Figure 8 based on SEM images. ZSM-5 shows small cubic crystals of around 200 nm, which form larger agglomerates of up to 12 μm (see Figure 8a). Kaolin consists of a layered structure without agglomerates (see Figure 8b).

Previous figures show that the starting materials have a different appearance and can therefore be distinguished in the formulated additive grains, as depicted in Figure 7. For example, the blue mark-up points out separated kaolin platelets, and the dotted red circles show cubic ZSM-5 crystals. While the molar content of ZSM-5 compared to kaolin is rather low (see Table 1), the total amount of ZSM-5 on the outer surface of the grain is relatively high. One origin is the different polarity of the compounds: with higher amounts of alumina binder, more polar components (ZSM-5) are visible on the external surface. With higher amounts of silica binder, there is a segregation of silica to the outer surface of the particle and of polar compounds in the inner volume of the catalyst grain.

**Figure 9:**
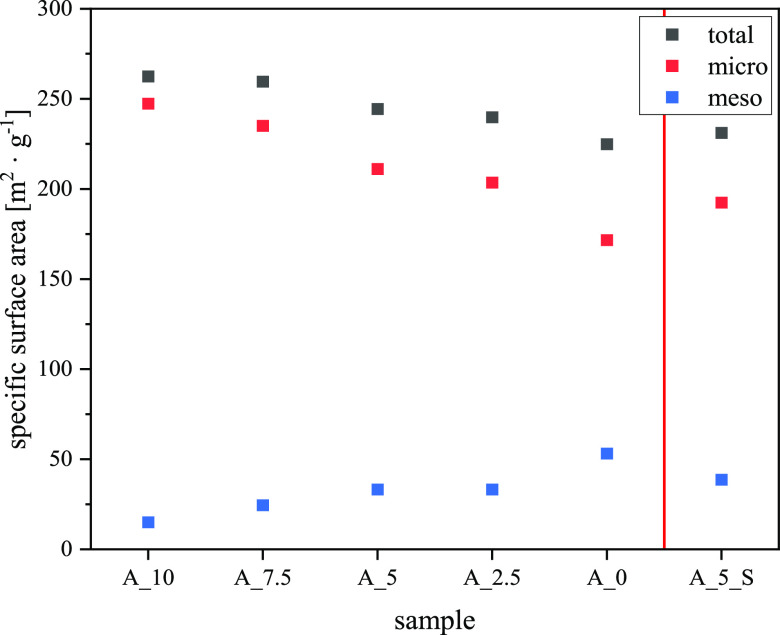
Influence of binder composition on the specific surface area from BET (microporous) and BJH (mesoporous) theories.

The elementary composition of the catalyst particles in Figure 6 additionally supports the statement that a high silica amount leads to a shell of binder around the catalyst particles. The smother appearance of samples with a high silica binder content supports this assumption. Therefore, physisorption isotherms with nitrogen were recorded, and surface areas caused by mesopores were calculated on the basis of BJH theory for further investigation (Figure 9).

**Figure 10:**
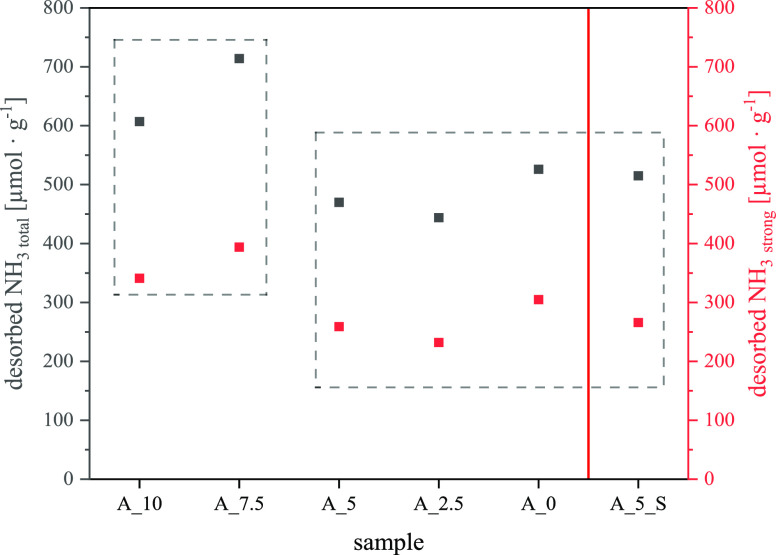
Calculated total acidities and acidities caused by strong acid sites from all samples; difference between two groups of a slightly higher acid site concentration (A_10, A_7.5) and a slightly lower concentration (A_5, A_2.5, A_0, A_5_S).

An increasing amount of LUDOX in the binder formulation leads to a decrease in the total specific surface area, in particular, to a decrease in the surface area caused by micropores (BET theory). In contrast, the specific surface area caused by mesopores increases with an increasing amount of the silica binder. These trends substantiate the idea of a capsulation of the catalyst particle by LUDOX. Due to this shell, a larger number of still accessible voids inside the particle is formed, which can be pictured by quantifying the mesopore volume and surface area. Contrarily, the alumina binder results in grains without shell or encapsulation after spray drying, which is the reason for the higher specific surface area caused by fully accessible micropores of the zeolite component. Besides the accessible surface area, the acid strength and concentration are of particular interest in explaining catalytic properties. For this, the calculated acidities from TPD measurements are depicted in Figure 10.

Figure 10 shows a weak correlation between the composition of the binder and the total amount of desorbed ammonia in TPD analysis and with respect to strong acid sites. Samples A_7.5 and A_10 show slightly higher TPD signals compared to the others. At higher amounts of LUDOX content, the total acid site concentrations as well as the number of strong acid sites are slightly reduced, especially in similar proportion. As strong acidity mainly originates from the active zeolite ZSM-5, the conclusion is a non-selective reduction of acid site concentration, for example, due to capping of the active zeolite particles (ZSM-5). This is a consensus on the results from the physisorption of nitrogen. However, small ammonia molecules easily access even narrow pores, which reduces the impact of morphology changes on the TPD signal.

**Figure 11:**
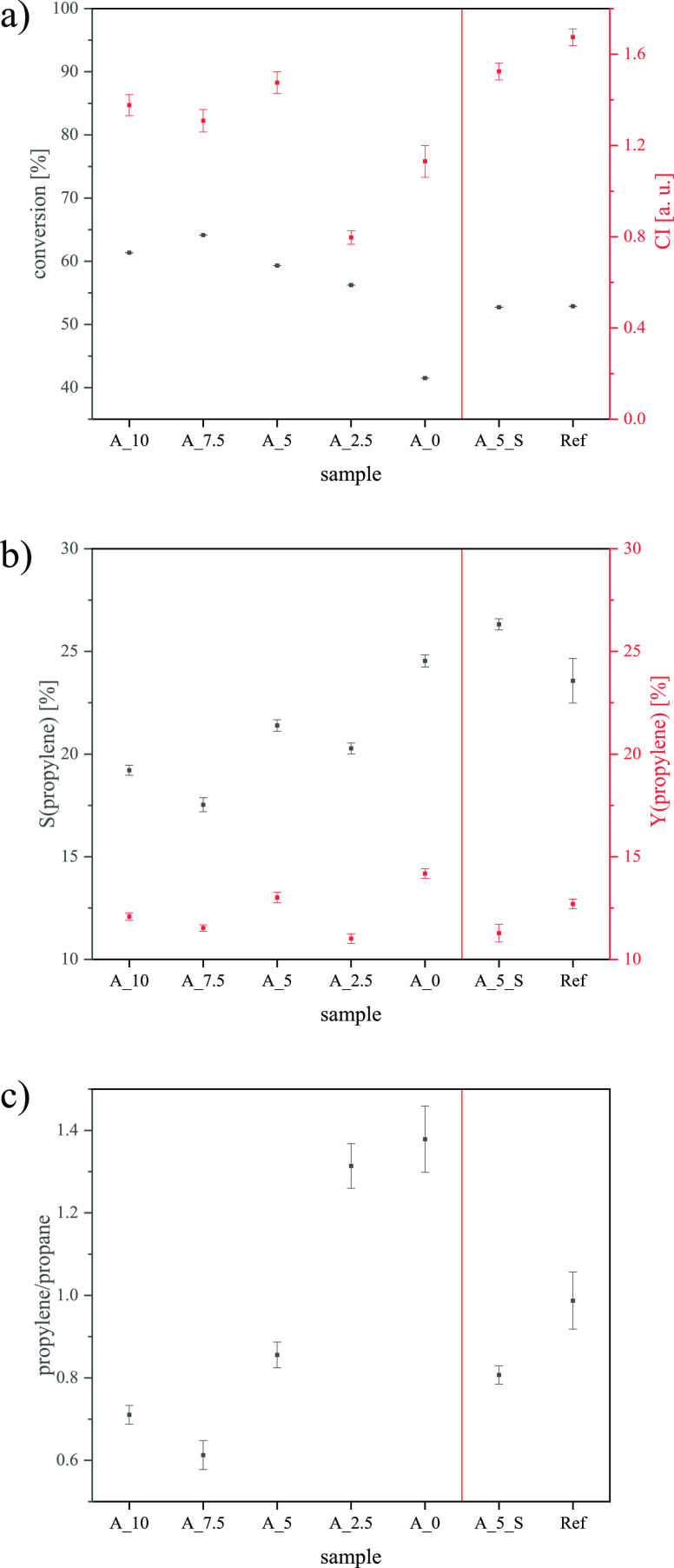
Results of catalytic tests for cracking a hexane mixture: (a) conversion and CI, (b) selectivities and yields of propylene, and the (c) ratio of propylene to propane.

The catalytic tests (see Figure 11 and Table 3) for cracking hexanes by prepared samples show comparable catalytic behavior to the used reference sample from Grace. While the kaolin filler remains almost inactive (see sample kaolin_A_10 in Table 3), different compositions of the binder lead to different catalytic properties. The decreasing amount of alumina leads to a decreasing conversion of the feed molecules (see Figure 11a). One reason could be that alumina species belong to an active matrix, while silica binders are usually part of the non-active matrix. Thus, a higher alumina amount leads to higher activity caused by additional acidity.^24^ With the addition of slight amounts of silica to the alumina binder, the Brønsted acid centers from alumina and zeolite are still accessible.^24,25^ This explains the comparable conversion of samples A_7.5 and A_10. However, the correlation between catalytic properties and TPD signals is weak due to easy access of ammonia to small and narrow pores. Even more prominent is the steady trend of the rising surface area with the alumina binder, which fits the trend of increasing hexanes conversion (see Figures 9 and 11a). Hence, the accessibility of active sites and the surface area for bigger molecules, such as hexane and their selective conversion, are more prominent parameters to analyze the behavior during cracking processes than ammonia TPD.

**Table 3:** Catalytic Performance of All Samples Considered: Conversion of Hexanes X, Constraint Index CI, Selectivity S, and Yield Y of Propylene

sample name	A_10	A_7.5	A_5	A_5_S	A_2.5	A_0	Ref	kaolin_A_10
X [%]	61.3 ± 0.1	64.2 ± 0.1	59.3 ± 0.1	52.7 ± 0.1	56.2 ± 0.1	41.5 ± 0.1	52.9 ± 0.1	2.1 ± 0.1
CI	1.4 ± 0.1	1.3 ± 0.1	1.5 ± 0.1	1.5 ± 0.1	0.8 ± 0.1	1.1 ± 0.1	1.7 ± 0.1	
S (C_3=_) [%]	19.2 ± 0.3	17.5 ± 0.3	21.4 ± 0.3	20.3 ± 0.3	24.5 ± 0.3	26.3 ± 0.3	23.6 ± 1.1	16.5 ± 0.4
Y (C_3=_) [%]	12.1 ± 0.2	11.5 ± 0.2	13.0 ± 0.3	11.0 ± 0.2	14.2 ± 0.2	11.3 ± 0.4	12.7 ± 0.2	0.5 ± 0.1

Since CI is a parameter describing the ratio of the conversion rates of different feed molecules, it can be used to evaluate the shape-selective cracking of *n*-hexane versus the more reactive 3-methylpentane. According to Frillette et al.^8^ ZSM-5 zeolite shows a CI ≫ 1 and CI < 12. This indicates a higher conversion of linear *n*-hexane over its bulkier branched isomer 3-methylpentane in narrow ZSM-5 pore channels of 0.58 × 0.52 nm,^8^ which can be observed for all samples except A_2.5. Compared to a commercial reference material, the CI values are lower, but there is still a higher cracking rate of *n*-hexane compared to that of 3-methylpentane. This confirms the shape-selective cracking property of the ZSM-5 component in the grains. However, samples A_0 and A_2.5 seem to lose this behavior due to the encapsulation of ZSM-5. Again, it reveals the site accessibility and the reactive surface as the main factors affecting the catalytic performance during the change in the binder composition.

**Figure 12:**
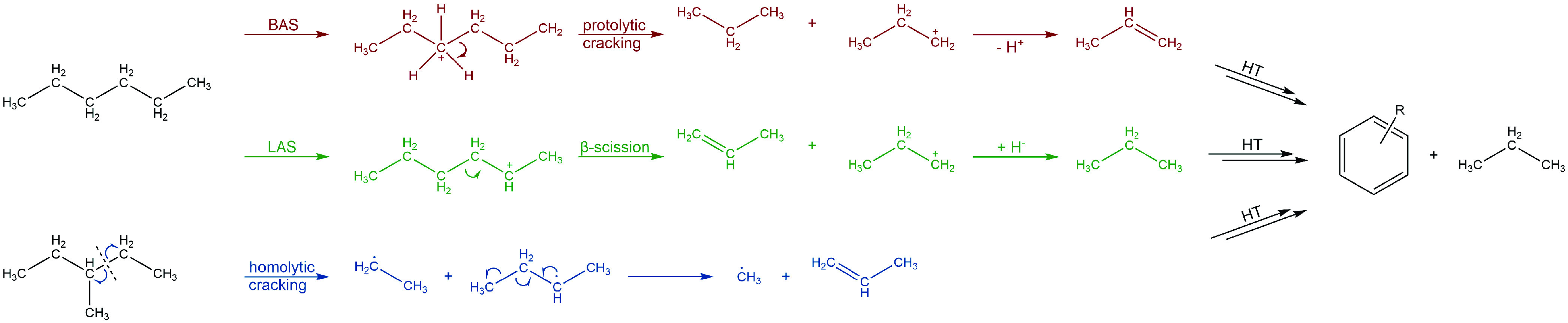
Formation of propylene and propane at BAS and LAS from starting materials *n*-hexane and 3-methylpentane up to secondary build-up products, for example, aromatics from hydrogen transfer (HT) reactions.

The selectivities for propylene and propylene yields (see Figure 11b and Table 3) depend on the conversion of the feed: at lower conversion rates for A_2.5 and A_0, the selectivity of propylene is higher, leading to a turning point of propylene selectivity for sample A_5. This also explains the shape of the graph in Figure 11c. The lower the conversion, the higher the selectivity for propylene and the higher the ratio of propylene to propane. In summary, propylene is an early cracking intermediate starting from hexane, which is formed at lower conversion or low residence times on the active surface. Propane (small alkanes) and aromatics are predominantly formed by secondary hydrogen transfer (HT) reactions, especially within the pore system of the active zeolite component ZSM-5. For a better understanding of the formation of C_3_ products, the reaction pathways are summarized in Figure 12.^2,26,27^ While Brønsted acid sites (BAS) predominantly promote the route of protolytic cracking via intermediary carbenium ions (red mechanism) and stoichiometric production of alkanes (e.g., propane), Lewis acid sites (LAS) also promote the direct formation of olefins and carbenium ions together, which enhances the olefin yield from cracking of hexanes (green mechanism), if secondary conversion processes are less dominant. While both support the conversion reactions, LAS show a higher preference for promoting hydride transfer reactions, which finally lead to coke. Although only minor amounts of coke have been observed during all tests (Table S3), an important impact of LAS on catalysis should also be considered because even small amounts of coke already affect pore accessibility.^6^ Especially at higher temperatures (>500 °C), there is an additional and significant contribution of homolytic cracking processes toward olefins (blue).

## 4 Conclusions

Different binder formulations of ZSM-5-containing cracking additives were investigated with a focus on their catalytic properties in the conversion of C_6_ hydrocarbon fractions. The results show that the performance strongly depends on the molar silicon to aluminum ratio of the binder. Alumina from aluminum chloride is part of the active matrix, while silicon dioxide is part of the inactive matrix.

Although the acidity of a pure alumina binder from AlCl_3_ is slightly higher than that of a pure silica binder as determined by ammonia desorption experiments, its correlation to acidity properties is weak. More importantly, the silica (LUDOX) used tends to form outer particle shells after spray drying, covering active and shape-selective ZSM-5, which confirms our previous studies.^28^ The investigations demonstrate that only small amounts of alumina in the mainly silica-based binder prevent the grain from shelling and ensure the access of hydrocarbon molecules to the active surface. The resulting good olefin yield in catalytic tests is due to an optimization between the suppression of excessive subsequent conversion steps after propylene formation from hexanes while still maintaining good accessibility of active sites and, thus, good activity. A Si/Al molar ratio between 5 and 6 in the binder was determined to be the optimal mixture; this composition (sample A_5) shows a high conversion rate as well as good selectivity toward the desired product propylene in the catalytic cracking of hexanes. Recent research reports show great interest in improving catalysts for cracking processes.^2,29^ Particular attention is paid to the different behaviors of binders not only for the control of porosity and topology but also to vary active site accessibility and introduce additional activity into cracking processes to fine-tune the intermediate alkanes and olefins formed.^2,29^

## Supporting Information Available

Temperature profile of TPD measurements; diffractograms of spray-dried additives (A_0—A_10) and reference materials (CBV (ZSM-5, CBV 5524G), and metakaolin); temperature profile of TPD measurements; temperature profile of the GC/FID method, modified ASTM D6729-14; coke content from TGA after 7 h ToS and mass fraction compared to the total mass balance including gaseous, liquid, and solid hydrocarbon products; and ζ-potential of sample slurries depending on the pH value (PDF)

## Abbreviations

3-MP: 3-methylpentane
BAS: Brønsted acid sites
BET: Brunauer–Emmett–Teller
BJH: Barrett–Joyner–Halenda
CI: constraint index
DHA: detailed hydrocarbon analysis
FID: flame ionization detector
GC: gas chromatography
GHSV: gas hourly space velocity
HT: hydrogen transfer
ICP-OES: inductively coupled plasma optical emission spectroscopy
LAS: Lewis acid sites
LHSV: liquid hourly space velocity
MTO: methanol to olefins
Ref: reference
SEM: scanning electron microscopy
TGA: thermogravimetric analysis
TPD: temperature-programmed desorption (of ammonia)
ToS: time on stream
XRD: X-ray diffraction
ZSM: Zeolite Socony Mobil
