# Supplementary material for: Crucial Role of Silica-Alumina Binder Mixtures for Hydrocarbon Cracking with ZSM-5 Additives
Source: ACS Omega. 2022 Nov 29;7(49):44892–902. doi: 10.1021/acsomega.2c05003 (PMC9753518; doi:10.1021/acsomega.2c05003)
Supplement: Supplementary file 1 — ao2c05003_si_001.pdf [file ao2c05003_si_001.pdf]

Supporting information

Crucial Role of Silica-Alumina Binder Mixtures for Hydrocarbon Cracking with ZSM-5 Additives

*Liane A. Haufe, Vladislav Timoshev, Markus Seifert, Oliver Busse, Jan J. Weigand**

AUTHOR INFORMATION

Corresponding Author

***Jan J. Weigand:** Technische Universität Dresden, Faculty of Chemistry and Food Chemistry, Chair of Inorganic Molecular Chemistry, Mommsenstraße 4, 01069 Dresden, Germany. Email address: jan.weigand@tu-dresden.de

Authors

Liane Andrea Haufe: Technische Universität Dresden, Faculty of Chemistry and Food Chemistry, Chair of Inorganic Molecular Chemistry, Mommsenstraße 4, 01069 Dresden, Germany.

Vladislav Timoshev: Technische Universität Dresden, Faculty of Chemistry and Food Chemistry, Chair of Inorganic Molecular Chemistry, Mommsenstraße 4, 01069 Dresden, Germany.

Markus Seifert: Technische Universität Dresden, Faculty of Chemistry and Food Chemistry, Chair of Inorganic Molecular Chemistry, Mommsenstraße 4, 01069 Dresden, Germany.

Oliver Busse: Technische Universität Dresden, Faculty of Chemistry and Food Chemistry, Chair of Inorganic Molecular Chemistry, Mommsenstraße 4, 01069 Dresden, Germany.

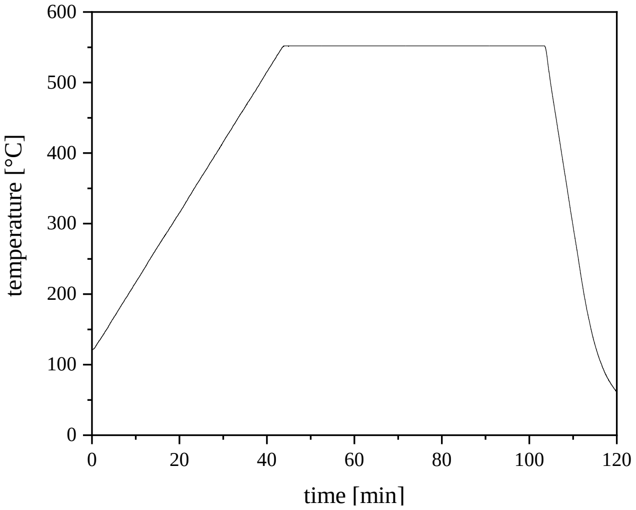

Figure S1: Time-dependent temperature profile of TPD measurements.

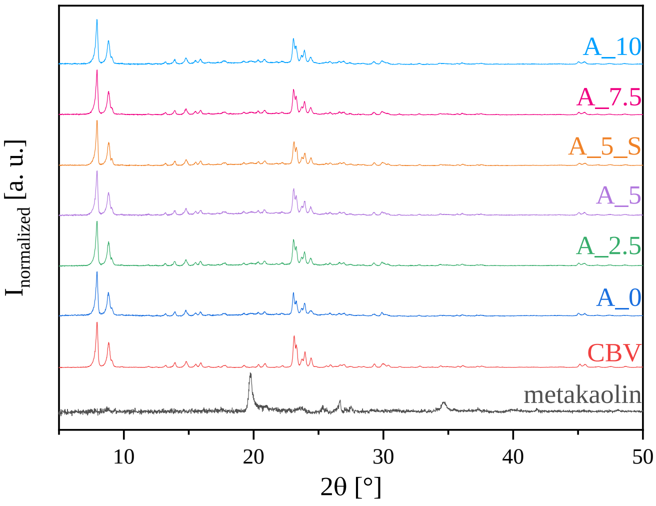

Figure S2: Diffractograms of spray-dried additives (A_0 – A_10) and reference materials (CBV (ZSM-5, CBV 5524G), metakaolin).

Table S1: Temperature profile of TPD measurements.

start temperature [°C]	heating rate [K·min^{-1}]	end temperature [°C]	hold time [min]
120	10	550	60

Table S2: Temperature profile of GC/FID method, modified ASTM D6729-14.[1]

start temperature [°C]	heating rate [$K \cdot min^{-1}$]	end temperature [°C]	hold time [min]
28	-	28	13
28	10	45	15
45	1	60	15

Table S3: Coke content from thermogravimetric analysis after 7 h ToS and mass fraction compared to the total mass balance including gaseous, liquid and solid hydrocarbon products.

	(left) coke fraction on catalyst [wt %] and (right) fraction to hydrocarbon mass balance [wt %]			
sample	test 1		test 2	
A_10	0.56	0.05	0.73	0.06
A_7.5	0.70	0.06	0.80	0.07
A_5	0.43	0.04	1.04	0.09
A_5_S	0.36	0.03	0.52	0.04
A_2.5	0.69	0.06	0.25	0.02
A_0	0.76	0.07	0.74	0.06
Ref	0.50	0.04	0.85	0.07
kaolin_A_10	0.64	0.06	-	-

Table S4: ζ-potential of sample slurries depending on pH value.

	ζ-potential [mV]			
pH	3	4	5	6
A_10	-6.09 ± 0.7	9.10 ± 0.6	29.80 ± 0.4	17.80 ± 0.7
A_7.5	-4.85 ± 0.3	6.82 ± 0.6	20.70 ± 2.2	20.90 ± 1.2
A_5	-5.88 ± 0.1	-3.69 ± 0.8	18.70 ± 1.4	24.40 ± 0.8
A_2.5	-8.27 ± 0.4	-5.81 ± 0.2	2.10 ± 0.2	-12.60 ± 0.6
A_0	-19.90 ± 0.2	-16.00 ± 0.5	-19.08 ± 0.3	-32.00 ± 2.0

REFERENCES

(1) ASTM International, Standard Test Method for Determination of Individual Components in Spark Ignition Engine Fuels by 100 – Meter Capillary High Resolution Gas, 2014, Beuth Verlag GmbH, Berlin (ASTM D6729-14).
